# Localized identification of seepage and ponding in earthen embankment using infrared thermography assimilated with different deep learning frameworks

**DOI:** 10.1038/s41598-025-13258-y

**Published:** 2025-10-15

**Authors:** Ritesh Kumar, Hans Henning Stutz, Kanupriya Johari

**Affiliations:** 1 Department of Earthquake Engineering, Joint Faculty at Center for Sustainable Energy, Indian Institute of Technology Roorkee, Roorkee, India; 2 Institute of Soil Mechanics and Rock Mechanics (IBF), Humboldt EVR Fellow at IBF KIT, Karlsruhe, Germany; 3 SPARK Intern, Department of Earthquake Engineering, Indian Institute of Technology Roorkee, Roorkee, India

**Keywords:** Earthen embankment, IR thermography, Health monitoring, Seepage, Ponding, ML and AI, Natural hazards, Engineering

## Abstract

Earthen embankments are built to prevent flooding and protect communities from the dangers of floods and high water levels. However, these geotechnical structures may not always remain serviceable and can fail due to long-term seepage and ponding. For instance, erosion causes the earthen structure to weaken and eventually fail, which may be due to several factors, including the velocity of the water, soil water characteristics, fine content, and gradation of the soil. The presented research explores an advanced approach to address the critical issue of identifying the seepage and ponding through the embankment by assimilating the passive infrared thermographic imageries with Deep Learning (DL) algorithms. To facilitate the development and validation of developed DL frameworks, a physical experimentation setup at the model scale is developed. This platform enabled the generation of a comprehensive dataset of thermal images across various environmental scenarios, including vegetation coverage and rainfall. Multiple DL frameworks were initially explored within the framework and the models were designed to process sequences of thermal images and predict the extent of seepage and ponding. This research builds upon effectively transforming the complex task of embankment leakage identification into an image classification problem. Moreover, the developed framework demonstrates that mapping of seepage and ponding can be achieved with great accuracy and is vital in enhancing embankment safety and disaster prevention strategies in flood-prone areas.

## Introduction

River embankments are critical flood control infrastructures that frequently suffer from seepage and ponding, particularly during flood seasons. The timely detection of these leaks is crucial, as unrecognized leakage can lead to catastrophic embankment breaches, resulting in widespread flooding, property destruction, and significant threats to public safety^1^. The history has witnessed several devastating consequences of embankment failure across the globe. For instance, a railway embankment in Southern Italy collapsed due to seepage following heavy rainfall^2^ in 2005. The 2019 Durgawati Dam in India faced seepage-induced slope failures^3^. Several other instances of embankment failure along the river Elbe in Eastern Germany have also been reported during floods due to seepage and ponding^4^. Moreover, nearly 1.6 million people were affected in the 2024 embankment breach in Bihar, India, underscoring the vital importance of early leakage detection in preventing such disasters^5^.

Conventional monitoring methods for leakage detection in reservoir dams, tailings, and levees; such as pressure gauges and weirs, are impractical for their extensive lengths. While geophysical prospecting techniques like resistivity detection^6^ and transient electromagnetic methods^7^ have been employed during non-flooding seasons, they lack the coverage, reliability, efficiency, and cost-effectiveness required for emergency detection during flood seasons. Even advanced techniques like optical-fiber-based distributed temperature monitoring systems face significant implementation challenges in existing embankments. Recent research has proposed various methods for ponding detection based on new equipment and platforms, including bionic dogs, manned vehicles, and ground monitoring equipment^8–13^. However, these methods are limited by factors such as range, cost, or size. In contrast, systems deployed to Unmanned Aerial Vehicle (UAV) platforms offer advantages in terms of size, weight, and cost-effectiveness.

In this regard, infrared thermal (IRT) imaging has proven to be an effective non-destructive method for detecting seepage in embankments by capturing surface temperature variations. As water seeps through an embankment, it alters the soil’s thermal properties due to its higher specific heat capacity, causing wet areas to retain heat longer during the day and cool down more gradually at night^14^. These temperature differences detected using IRT images, help identify potential seepage zones. Recent studies have demonstrated the effectiveness of UAV-mounted IRT for monitoring levee seepage, allowing for efficient large-scale surveys^15^. Numerical modeling also supports its ability to detect seepage in dams by analyzing temperature anomalies. Compared to conventional methods such as piezometers and borehole investigations, which are invasive and expensive, IRT images provide a fast, cost-effective, and non-intrusive alternative. Its capability to inspect large and inaccessible embankments enhances its applicability^16^. Additionally, integrating IRT with UAV technology facilitates real-time monitoring, enabling early intervention before severe damage occurs. Long-term thermal monitoring has been shown to predict seepage-related instabilities, reducing risks of failure and maintenance costs^16,17^. Moreover, by utilizing temperature-based detection and UAV advancements, IRT with appropriate resolutions and thermal sensitivity (discussed later in Section 2) is becoming a key tool in geotechnical engineering for proactive seepage assessment.

The testing setup equipped with visible light and thermal infrared detection systems can perform near-real-time detection and processing, enabling rapid and large-scale inspection of embankment failures^18^. The challenge, however, lies in the vast amount of thermal data collected in this process, which necessitates the development of automatic identification methods for leakage targets. Zhou et al.^19^ pioneered the use of AlexNet to transform the issue into an image classification problem for leakage detection. Building on a similar foundation, our study aims to determine the accurate position of leakage based on predicted values and optimize the process using advanced deep-learning networks. By addressing the limitations of current inspection methods and harnessing the power of advanced imaging and machine learning technologies, this research aims to significantly enhance the ability to detect and respond to embankment seepage. The outcomes of this study have the potential to improve flood control measures and public safety in flood-prone areas by providing a more reliable and efficient solution for detecting the seepage and ponding anomalies leading to embankment failure. In the presented research, a physical setup is developed to simulate seepage and ponding through the embankment replicating real-world embankment conditions. This platform enables the generation of a comprehensive dataset of infrared images depicting various seepage and ponding scenarios. These images are then used to train and fine-tune state-of-the-art deep convolutional neural networks. To validate the practicality, robustness, and generalization capabilities of the developed framework, extensive testing within different environmental condition scenario was also carried out.

## Methodology

This study employs a systematic research methodology comprising several key phases to address the challenge of automatic identification of river embankment seepage and ponding. In the first phase, a sophisticated physical setup was developed to conduct the experiments. The novel setup was equipped with the facilities to replicate seepage and ponding mechanism as discussed in the next subsection in detail. Moreover, this platform served as a controlled setting for generating diverse leakage scenarios, enabling the collection of a comprehensive dataset of infrared images capturing various seepage-induced thermal anomalies in the second phase. In the third phase, leveraging this rich dataset, the authors developed state-of-the-art deep learning models trained to identify thermal anomalies associated with embankment leakage automatically. The presented framework effectively transforms the complex task of leakage detection into a more tractable problem of thermal anomaly recognition in infrared imagery. Thermal anomalies show a strong correlation with actual leakage occurrences, making them a reliable indicator for detection. The trained models leverage these thermal signatures to identify leaks effectively, offering a non-invasive and efficient approach.

### Development of a physical experimental setup

**Fig. 1:**
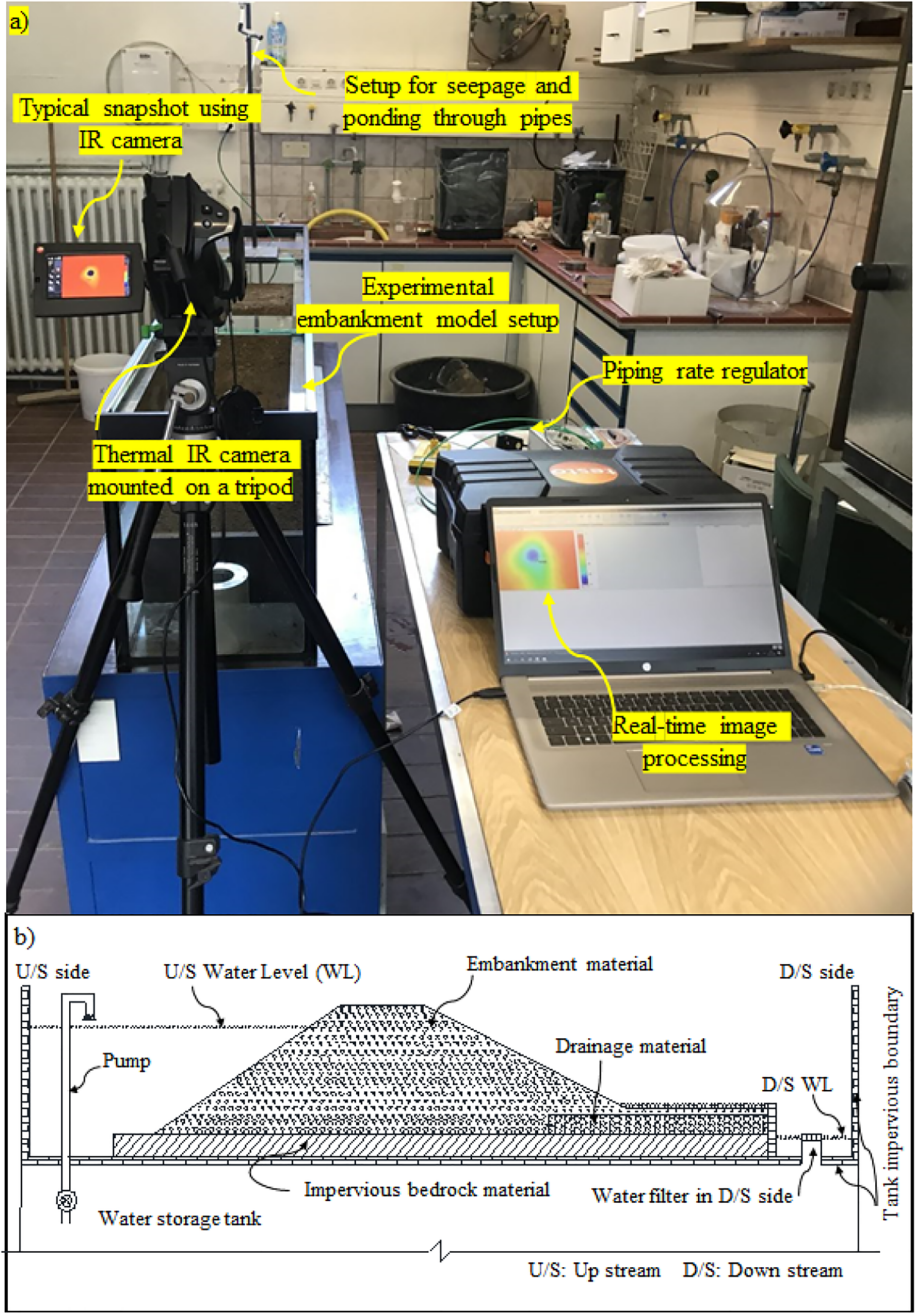
A comprehensive arrangement of the physical experimental setup: (**a**) typical layout of all accessories involved in the experiment, (**b**) a typical schematic layout of embankment model.

A physical setup was developed in-house to conduct experiments on embankment seepage and ponding consisting of two major components: an embankment modeling section and an arrangement for controlling seepage flow as shown in Fig. 1. The embankment model was housed in a top container made of transparent Plexiglas/acrylic sheets, ensuring visual monitoring, with arrangements to prevent bulging of the side sheets. This container was placed within a primary tank constructed from aluminum sheets, providing structural stability. The tank had impervious sides and a bottom to prevent leakage, while filters were installed at the outlet to facilitate proper drainage. The second component included a system for generating and maintaining the required pressure head using a suitable pump, with strategically placed inlets and outlets ensuring zero disturbance to the embankment model. Additionally, provisions were made to regulate the downstream water level, allowing for a stable phreatic surface. The setup ensured continuous water flow through the embankment once steady-state conditions were achieved, enabling controlled experimentation on seepage and ponding mechanisms. Several other accessories were also integrated, including a thermal camera, a ponding setup, a ponding and seepage rate regulator, a rainfall simulator, and a real-time image processing system, which are discussed in the next subsections.

### Embankment modeling scheme

**Fig. 2:**
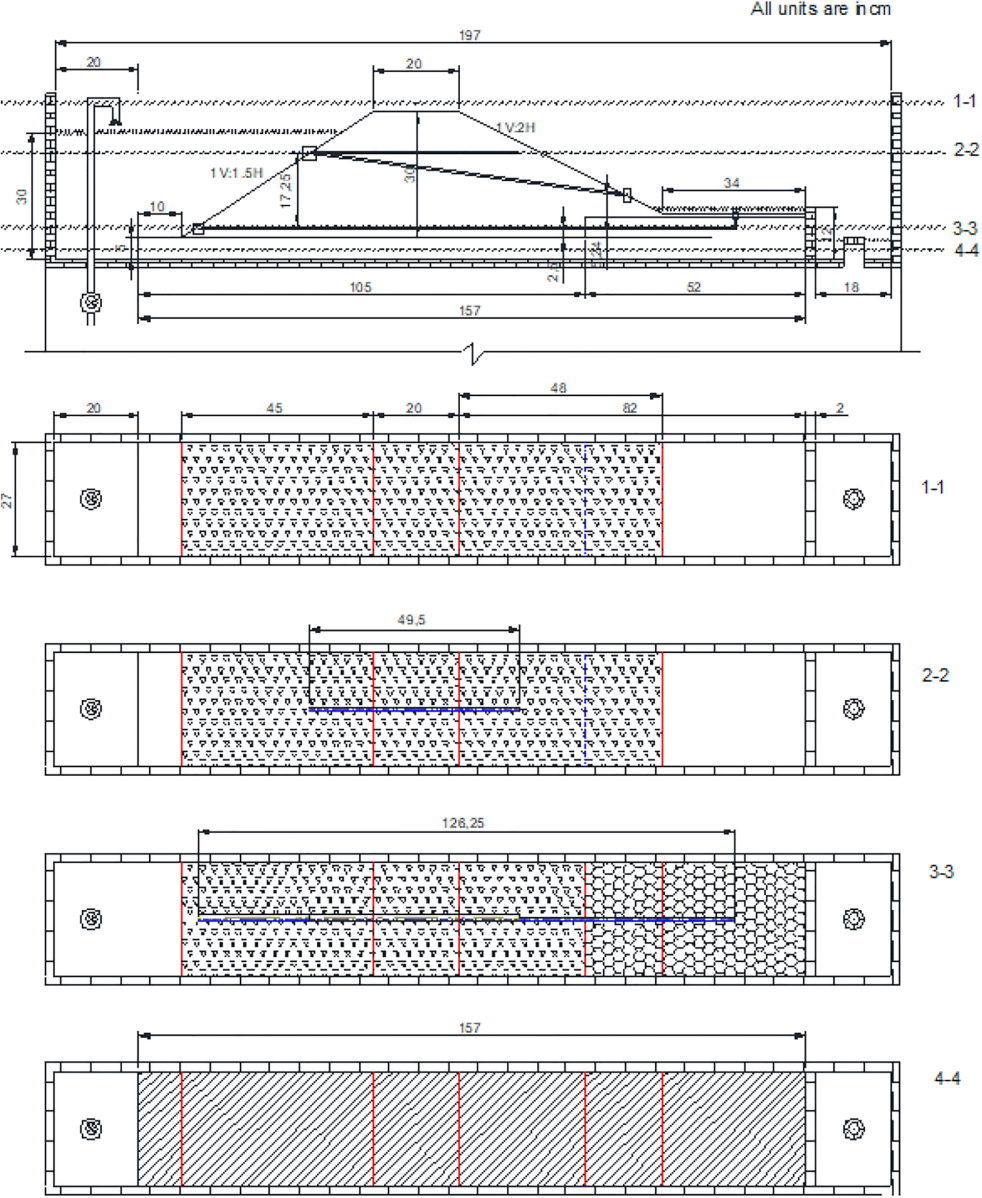
Schematic layout of embankment model with all dimensions along with the top view of different cross sections and arrangements for seepage and ponding mechanism. The ponding is achieved with the help of the bottom-most pipe.

**Fig. 3:**
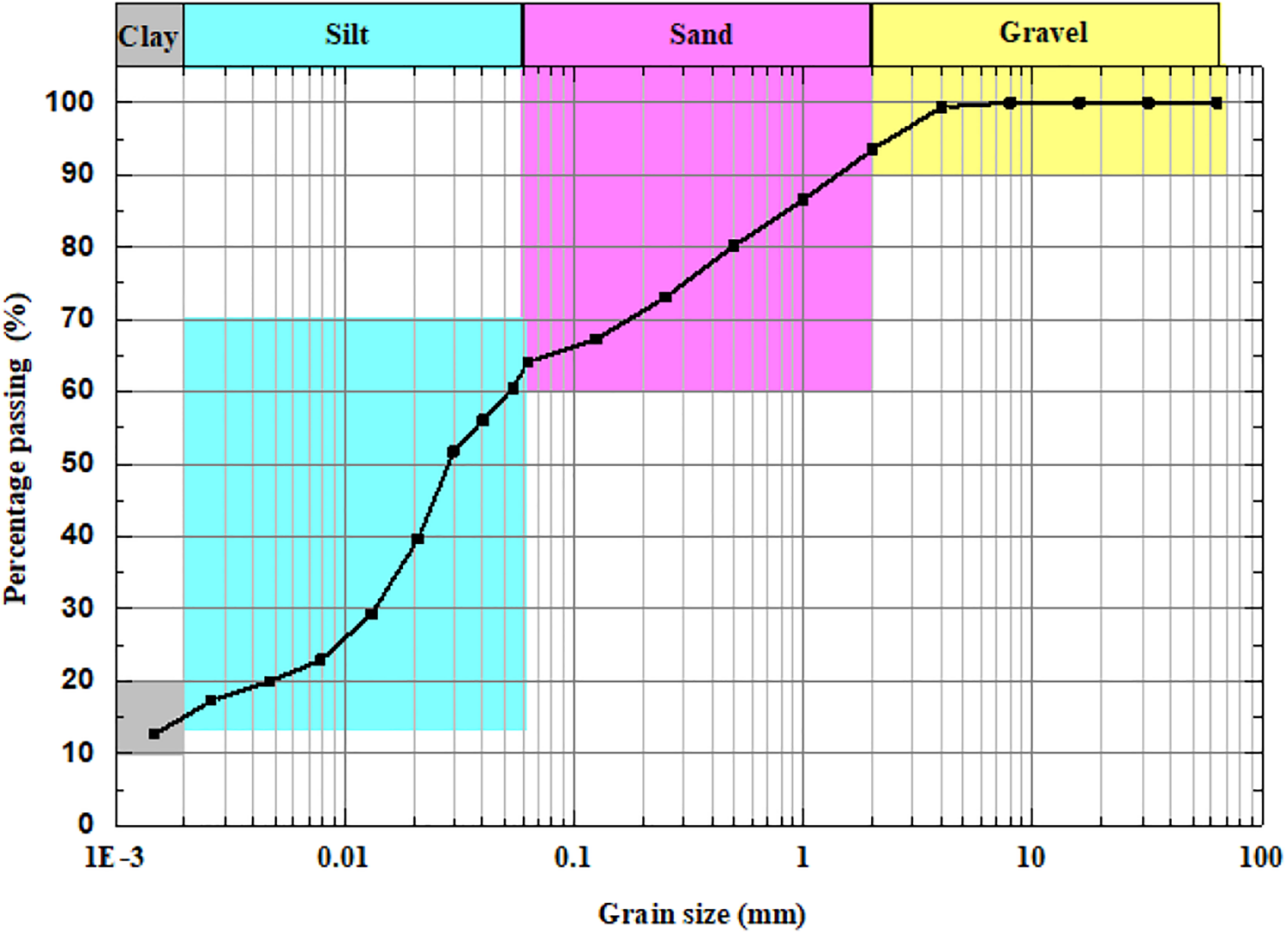
Grain size distribution of embankment material.

A detailed schematic layout of the embankment model is shown in Fig. 2, with all dimensions in centimeters unless otherwise stated. The embankment was constructed using the homogenized silty soil sourced from MinERALiX GmbH in Germany, and its grain size distribution is presented in Fig. 3. The upstream (U/S) and downstream (D/S) slopes of the embankment were maintained at 1V:1.5H and 1V:2H, respectively, ensuring a stable geometry. To facilitate drainage, Silica No. 3 was used, while a Plexiglas sheet was placed at the base of the model to simulate an impervious bedrock, as depicted earlier in Fig. 1. The index properties of both drainage material and embankment material is tabulated in Table 1. The index properties of the used materials align with typical construction materials of aged or poorly designed embankment materials across the world^20–22^. The embankment model was constructed layer by layer, with each layer being compacted through soft tamping to achieve the required density. The layers were built in 5 cm increments, ensuring uniformity and minimizing disturbances to the model. Guide plates were strategically positioned to maintain smooth slopes, and any excess soil was carefully slid with minimal disturbance to the embankment model. To study the effects of seepage and ponding, three pipes, each with a diameter of two mm, were embedded within the embankment at specific locations during construction as shown in Fig. 2. The bottom pipe was positioned to simulate ponding beneath the foundation of the embankment, allowing for the study of ponding-induced failure mechanisms at the base. The inlets of the top two pipes were placed at a height of 17.25 cm (in model scale) above the embankment base, with their respective outlets oriented at different angles to replicate varying seepage flow paths. It is important to note that the outlets of all three pipes were not fully exposed on the downstream side, as they remained embedded within the embankment by 1 5 cm, ensuring actual seepage and ponding conditions. Additionally, the pipe inlets were carefully wrapped with a very soft fabric to prevent soil particles from entering the pipes while allowing water to pass through freely. It is to be noted that the ensuring a natural ponding or seepage induced leakage is neither feasible nor time effective in a small experimental setup. Therefore, novel synthetic arrangements are made in the model itself to mimic the real field scenario.

To provide a comprehensive view of the embankment and pipe placements, four different cross-sections were illustrated in Fig. 2, offering insights into the top-down perspective of the embankment model during its preparation. These cross-sections aid in visualizing the spatial distribution of the pipes and embankment layers, ensuring clarity in understanding the experimental setup. The systematic construction approach, combined with precise pipe placements, ensures that the embankment model accurately replicates real-world seepage and ponding phenomena.

**Table 1:** Index properties of drainage and embankment material^23^.

Description	Index properties	
	Drainage material	Embankment material
Specific gravity, $G_s$	2.6	−
$D_{50}$ (mm)	1.72	0.027
$D_{10}$ (mm)	1.37	0.001
Maximum void ration, $e_{max}$	1.009	−
Minimum void ration, $e_{min}$	0.697	−
Permeability, k (m/s)	$6.6E^{-3}$	−
Approximate relative density, $D_r$	$45\%$	$90\%$
Liquid limit	−	$30.7\%$
Plastic limit	−	$20.6\%$

### Testing scheme for acquisition of IR images of embankment leakage and ponding

A sophisticated thermographic camera, the Testo 890, was used to capture thermal imageries throughout the experiment. The Testo 890 is a high-precision infrared camera equipped with a detector resolution of 640 $\times$ 480 pixels, enabling detailed thermal mapping. It features a thermal sensitivity of <40 mK, allowing it to detect minimal temperature variations, making it suitable for seepage and ponding studies. The camera operates within a spectral range of 7.5 to 14 $\upmu \text {m}$, ensuring accurate infrared detection. Its integrated resolution technology enhances image resolution, and a rotatable display facilitates ease of use in field conditions. Detailed specifications of the Testo 890 infrared camera are tabulated in Table 2 for ready reference. Thermal imageries were captured under various testing conditions, as tabulated in Table 3. In all test cases, thermal imaging was used to monitor both seepage-induced leakage and ponding signatures, helping to identify variations in subsurface water movement and associated thermal anomalies.

**Fig. 4:**
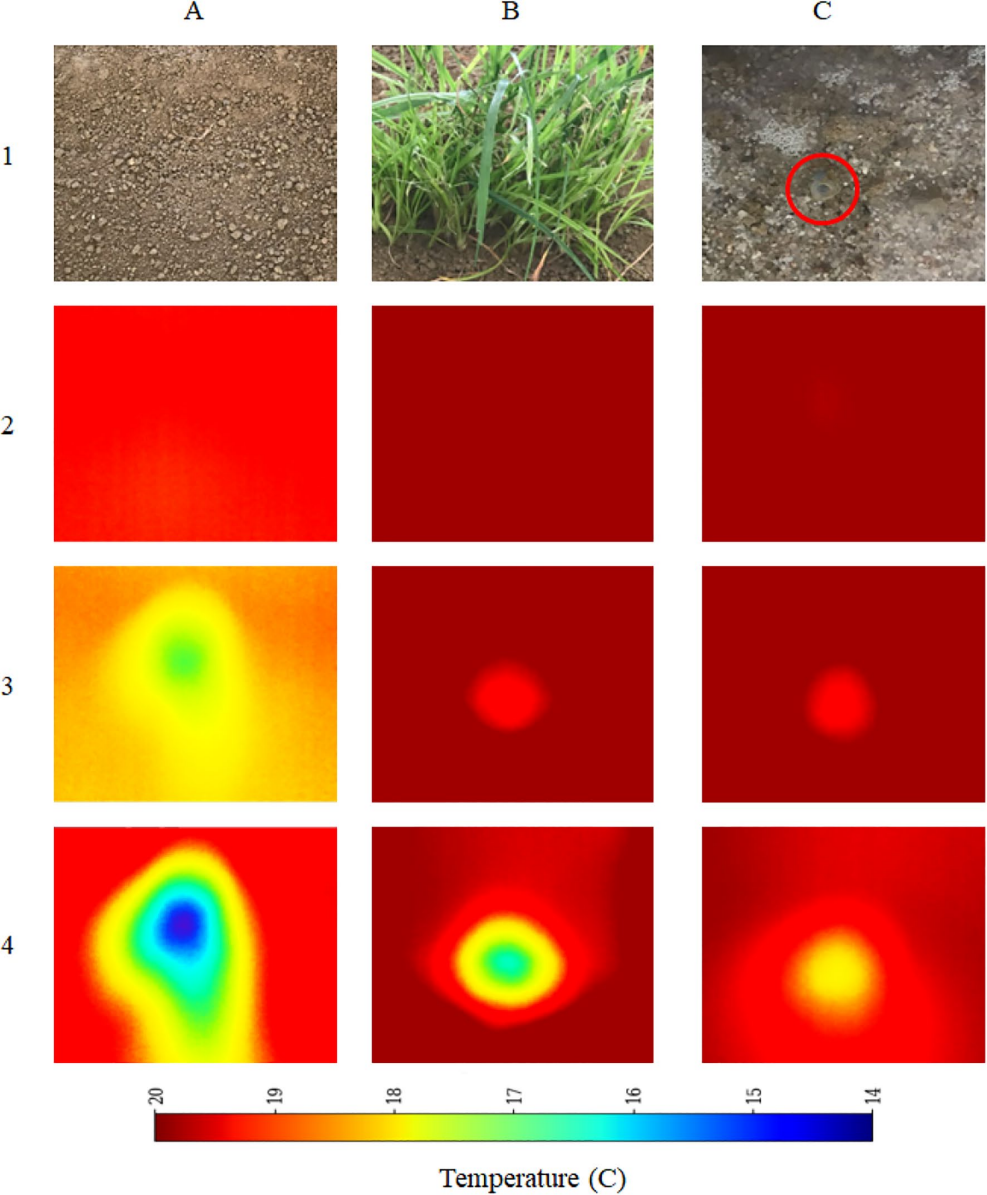
Conventional (RGB) and thermal images captured under different experimental conditions and at various stages of seepage and ponding. Tags A1 C4 represent three scenarios: clean dry surface, dry vegetation, and ponding (pipe circled). Row 1: Conventional image; Rows 2 4: Thermal images showing pre-seepage, onset, and after prolonged seepage/piping, respectively.

Ice was utilized to reduce the temperature of the water, which was subsequently circulated through the designated piping system (refer to Figure 2) to simulate seepage and ponding conditions. The temperature difference between the ambient environment and the embankment material—whose properties are summarized in Table 1 was maintained within a range of 0 to 7 °C across various test scenarios, as tabulated in Table 3. Figure 4 presents both conventional (RGB) and thermal images captured under different experimental conditions and at various stages of seepage and ponding. In this figure, tags A1 A4, B1 B4, and C1 C4 correspond to scenarios representing: dry and clean surfaces, dry vegetation, and clear ponding, respectively. For enhanced clarity, the pipe associated with ponding is enclosed in a circle. The first row (tag 1) in each group (A C) displays the conventional images. The subsequent rows (tags 2 4) depict thermal images. Specifically, row 2 captures the thermal state before the onset of the seepage or ponding, row 3 shows the condition immediately following the onset of seepage or ponding, and row 4 illustrates the condition after prolonged seepage and piping. For consistency, a fixed temperature legend is also included in Figure 4.

The working principle of thermal imaging in detecting seepage and ponding is based on the difference in thermal properties of soil and water^17,24^. The specific heat capacity of soil is typically lower than that of water, meaning soil heats up and cools down more rapidly than water. The specific heat capacity of dry soil ranges usually from 0.8 to 1.3 kJ/kg$\cdot$K, whereas water has a significantly higher value of 4.18 kJ/kg$\cdot$K. Due to this difference, water-saturated zones within the embankment retain heat longer or take more time to heat up, leading to detectable thermal gradients^25,26^.

Infrared thermography relies on the principle of thermal radiation, which states that all objects emit electromagnetic waves according to their temperature. Planck’s law describes the spectral radiance $L(\lambda , T)$ of a blackbody as^27^:

1 $$\begin{aligned} L(\lambda , T) = \frac{2hc^2}{\lambda ^5} \frac{1}{e^{(hc/\lambda kT)} - 1} \end{aligned}$$

where: $h$ is Planck’s constant ($6.626 \times 10^{-34}$ J$\cdot$s), $c$ is the speed of light ($3.0 \times 10^8$ m/s), $k$ is the Boltzmann constant ($1.38 \times 10^{-23}$ J/K), $\lambda$ is the wavelength of emitted radiation, and $T$ is the absolute temperature in Kelvin. The emissivity of soil and water also influences thermal imaging results. The emissivity of dry soil varies between 0.90 and 0.95, while water has an emissivity close to 0.98. Since water retains heat longer, seepage-affected regions appear as thermal anomalies in infrared imagery. By capturing continuous thermal data, the temperature distribution of an embankment can be analyzed to distinguish leakage patterns and identify potential failure zones. This technique provides a non-intrusive method for monitoring ponding and seepage dynamics, improving early detection of ponding failures.

**Table 2:** Technical specification of Testo 890 thermal camera used in the experiments.

Technical specification	Reliability
Field of view	$42^{\circ }$ x $32^{\circ }$ (Standard lens), $25^{\circ }$ x $19^{\circ }$ ($25^{\circ }$ lens), $15^{\circ }\times 11^{\circ }$ (Telephoto lens), $6.6^{\circ }\times 5^{\circ }$ (Supertele)
Minimum focus distance	0.1 m (Standard lens), 0.2 m ($25^{\circ }$ lens), 0.5 m (Telephoto lens), 2 m (Supertele)
Geometric resolution	1.13 mrad (Standard lens), 0.68 mrad ($25^{\circ }$ lens), 0.42 (Telephoto lens), 0.18 (Supertele)
Infrared resolution	640 x 480 pixels
Thermal sensitivity	< 40 mK at +$30\ ^{\circ }\text {C}$
Spectral range	7.5 to 14 $\upmu \text {m}$

**Table 3:** Number of thermal images collected for different test conditions on D/S side.

Testing condition	Number of thermal images
Rainfall over vegetation	118
Dry vegetation	120
Dry and clean surface	300
Rainfall over clean surface	131
Clear ponding	230
Ponding under rainfall	245

###  Referencing of the temperature and integration of Deep Learning methodologies

Different learning-based approaches for detecting seepage and ponding through thermal image sequences are discussed in this subsection. To ensure data consistency, a standardized preprocessing pipeline was applied, including conversion from BMT to PNG (either manually or automatically), color transformation from BGR to RGB, resizing to $224\times 224$ pixels, and normalization of pixel values to the range [0, 1]. These steps ensure uniformity across datasets, minimizing inconsistencies in model input.

Thermal infrared imaging captures temperature variations influenced by the thermal properties of different materials^28^. Since leakage zones generally exhibit lower temperatures than their surroundings, precise temperature mapping is crucial to mitigate false color distortions. To achieve this, a calibrated temperature transformation function is applied:

2 $$\begin{aligned} T(x,y) = T_{min} + \frac{H(x,y)}{H_{max}}(T_{max} - T_{min}) \end{aligned}$$

where *H*(*x*, *y*) represents the hue value at pixel (*x*, *y*), $H_{max}$ is the maximum hue value, and $[T_{min}, T_{max}]$ defines the expected temperature range. Anomaly detection is performed by computing the temperature difference $\Delta T_{i,j}$ between adjacent pixels using Equation (3):

3 $$\begin{aligned} \Delta T_{i,j} = |T(i,j) - T(i+1,j+1)| \end{aligned}$$

**Fig. 5:**
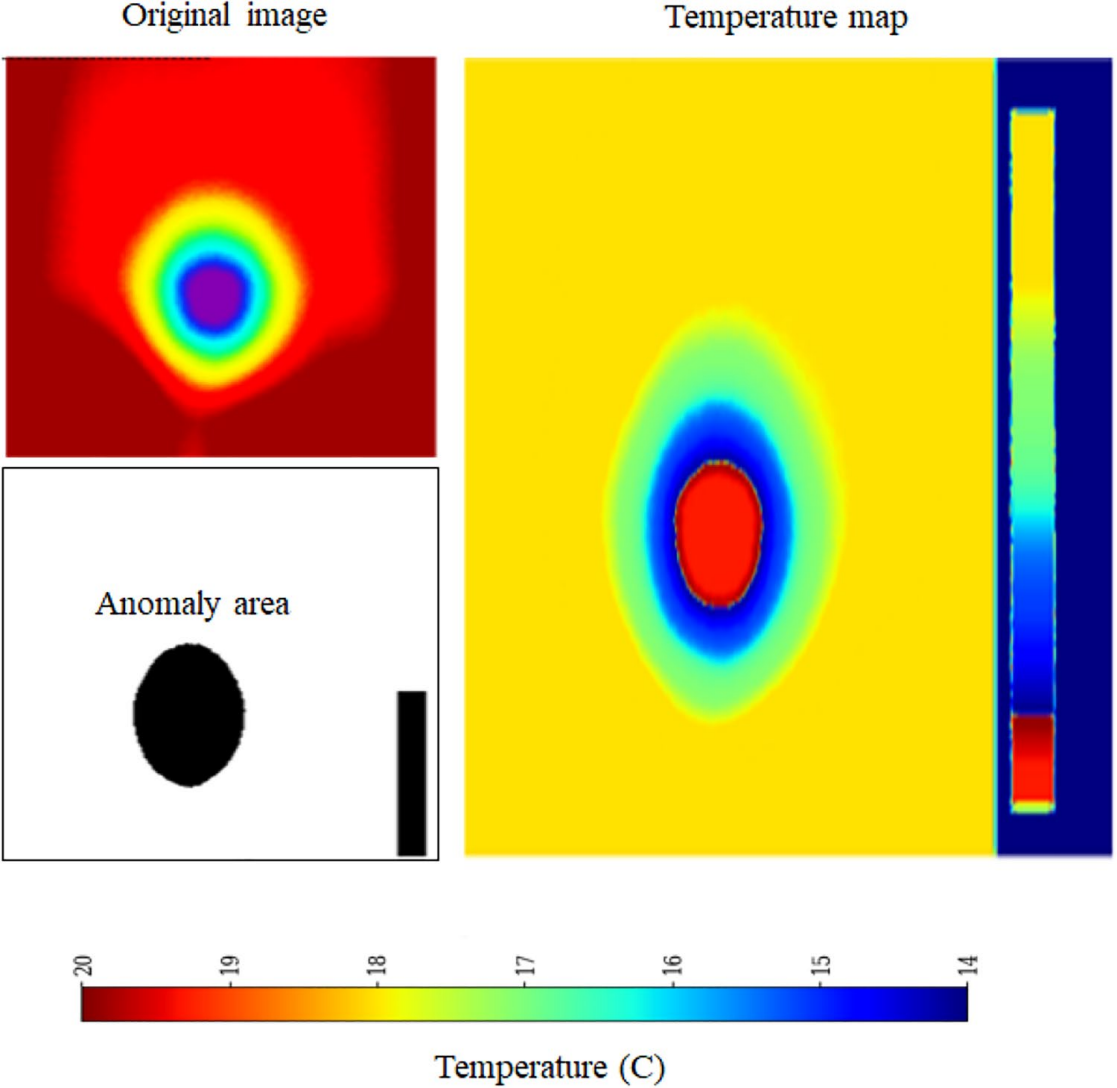
Pictorial presentation t of leakage detection algorithm.

The leakage detection algorithm, graphically outlined in Figure 5, applies an empirically determined threshold $\tau$ (typically $1^{\circ }\text {C}$) to filter out minor variations while preserving significant temperature anomalies indicative of leakage. Each image frame is regarded as a complete reference (100$\%$ pixel coverage), with leakage areas quantified by the proportion of pixels displaying significant temperature variations as outlined above. Pixels satisfying $\Delta T_{i,j}> \tau$ are classified as potential leakage points, as abrupt thermal fluctuations typically correspond to seepage or pipeline failures. To capture temporal dependencies, the framework primarily employs five-frame sequences (Seq5), balancing short-term anomaly detection with computational efficiency. Additionally, ten-frame sequences (Seq10) were also tested exclusively with the EfficientNet-LSTM model, as longer sequences led to performance degradation in other DL frameworks. By enforcing anomaly consistency across frames, the sequence-based approach minimizes false positives and enhances early-stage leak detection^29,30^.

Each DL framework processes image sequences of shape (sequence_length: 224, 224, 3) along with one-hot encoded environmental conditions. Feature extraction is performed using a time-distributed Convolutional Neural Network (CNN), ensuring spatial feature learning across frames. The extracted representations are processed by two stacked Long Short-Term Memory (LSTM) layers, capturing temporal dependencies. The LSTM output is concatenated with environmental features before being passed through fully connected dense layers, which refine learned representations for final leakage prediction. This architecture effectively integrates spatial, temporal, and environmental information, optimizing predictive accuracy. Leakage severity is quantified as the percentage of pixels with temperature anomalies exceeding the threshold, providing a continuous assessment rather than binary classification. To enhance contextual adaptation, environmental conditions (wet grass, dry grass, manual watering, and rainfall) are encoded as one-hot vectors and integrated into the model’s prediction layers. This multi-modal approach enables the network to adjust its outputs based on external factors influencing thermal signatures in outdoor environments. To identify the most effective leakage detection framework, four neural network architectures were evaluated: EfficientNetB0, AlexNet, ResNet, and CNN-LSTM. Each framework was trained following a standardized methodology incorporating domain-specific enhancements^31,32^. The evaluation metric, optimization strategy, loss function, performance monitoring, and transfer learning technique is described in the next subsection for each DL framework with the help of algorithms.

## Results and discussion

The architecture and quantitative performance of each deep learning (DL) framework are examined in terms of prediction accuracy relative to actual leakage, along with the mean absolute errors (MAE) across different epochs. To ensure robustness, all images were systematically compiled from various testing scenarios, as detailed in Table 3. This strategy was employed to minimize potential biases introduced during the training phase due to specific testing conditions, thereby enhancing the reliability of the proposed framework. A comparative analysis of the developed DL models under different testing conditions is also provided in the following subsection for clarity and conciseness.

### Performance of EfficientNet-B0 framework

EfficientNet is a convolutional neural network architecture and scaling method that uniformly scales all dimensions of depth/width/resolution using a compound coefficient, proposed by Tan and Le^33^. It has achieved state-of-the-art accuracy on ImageNet while being significantly smaller and faster than previous models^33^. The proposed framework is implemented using a structured deep-learning approach. It employs the EfficientNetB0 architecture as the feature extractor, utilizing pre-trained ImageNet weights while omitting the top layers. To handle sequential image data, a time-distributed EfficientNetB0 is incorporated, ensuring effective spatial feature extraction across frames. The extracted features are then processed through two Long Short-Term Memory (LSTM) layers, comprising 128 and 64 units, respectively, to capture temporal dependencies. Following this, a fully connected dense layer with 128 units and ReLU activation refines the learned representations. Finally, the model outputs a single-unit dense layer with a sigmoid activation function, scaling predictions within the range of 0 to 100%. This architecture enables robust feature extraction and sequence modeling, optimizing performance for image-based temporal analysis.

**Fig. 6:**
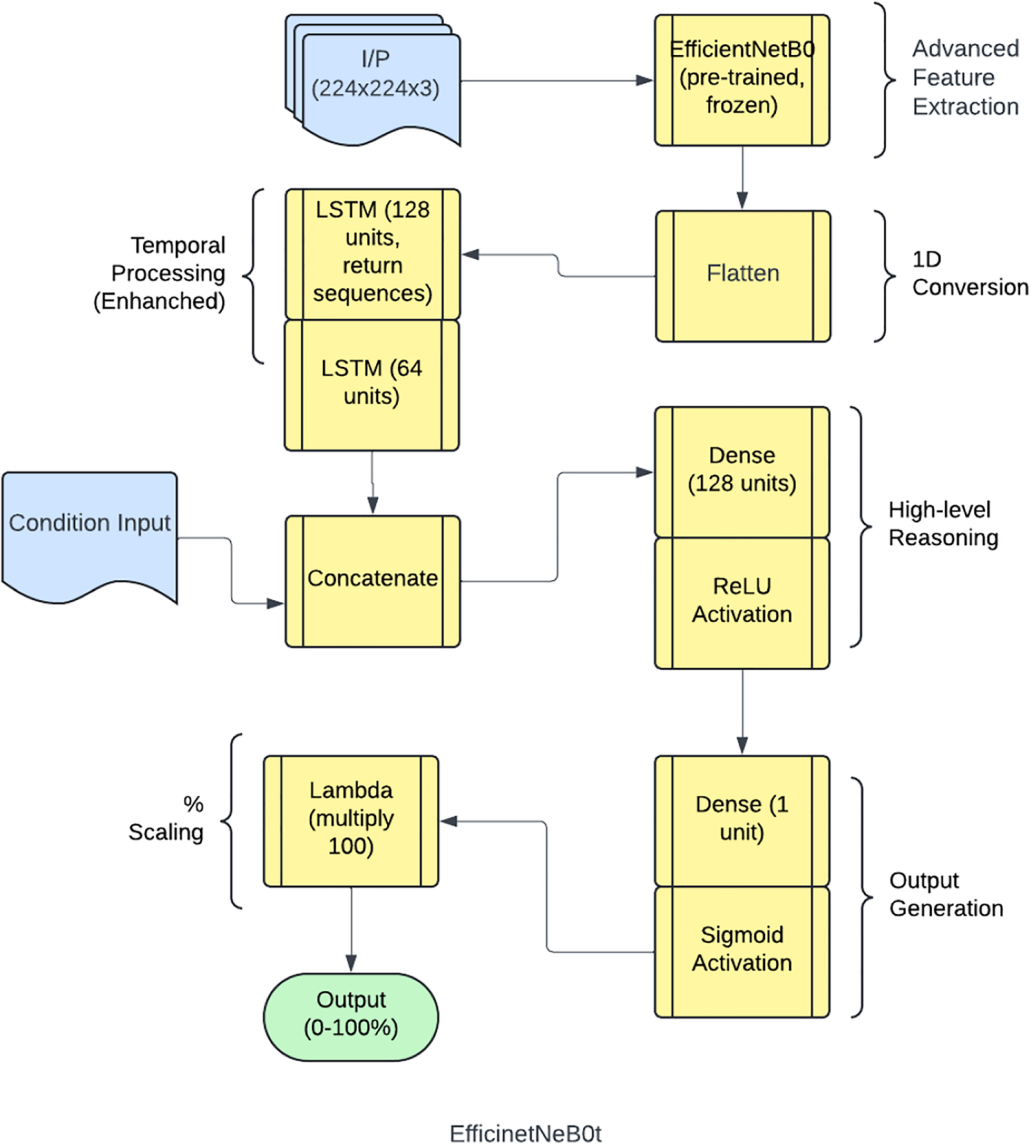
Architecture of the EfficientNet-B0 framework.

**Fig. 7:**
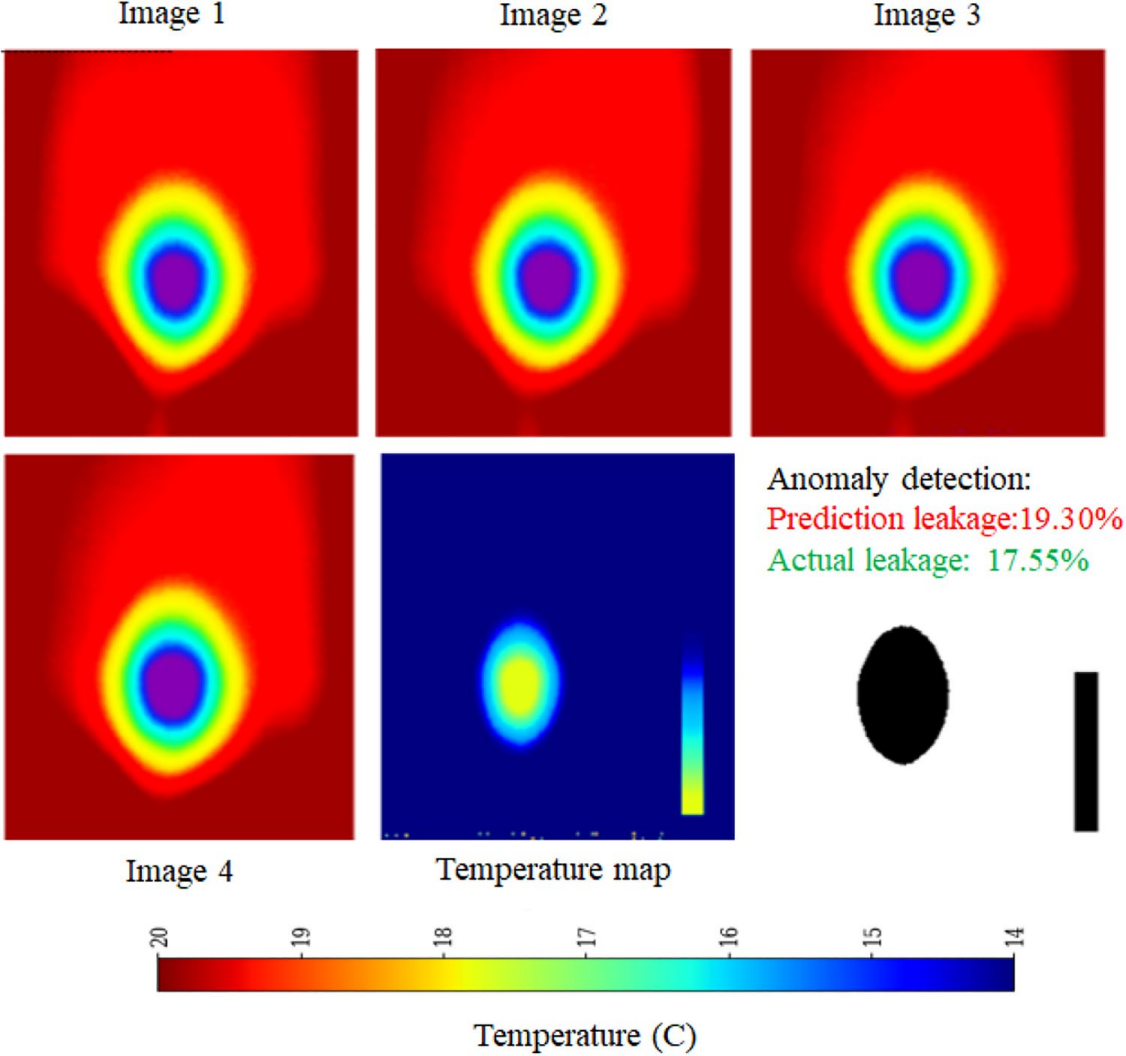
Performance of EfficientNet Seq5 framework.

**Fig. 8:**
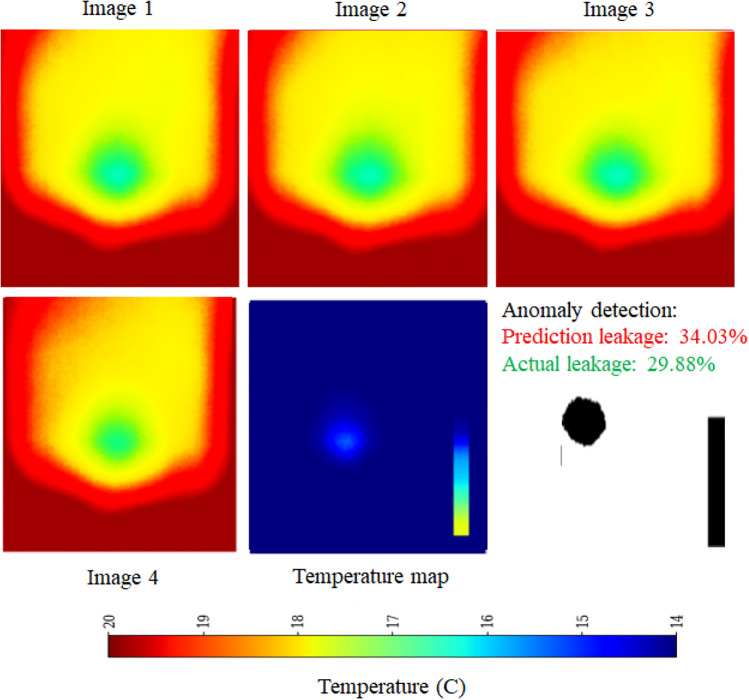
Performance of EfficientNet Seq10 framework.

**Fig. 9:**
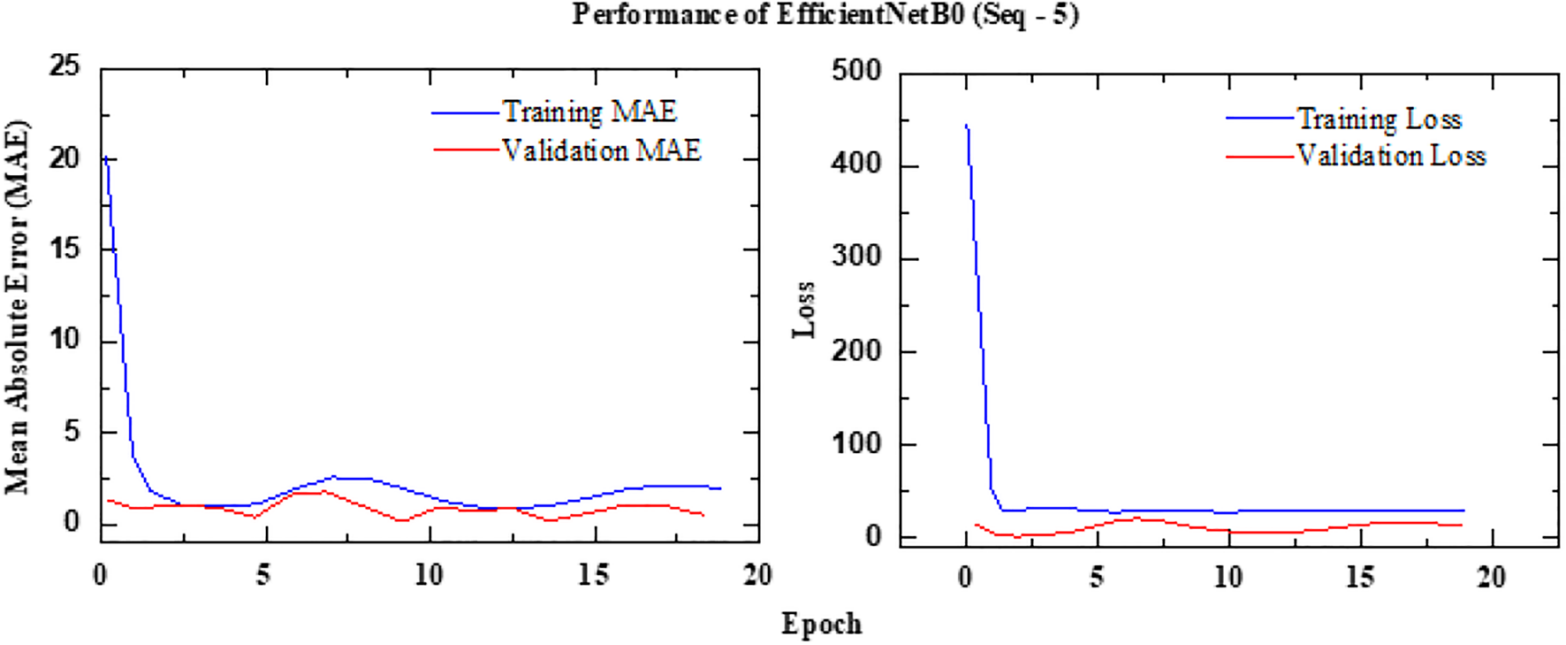
MAE Performance of EfficientNet Seq5 framework.

**Fig. 10:**
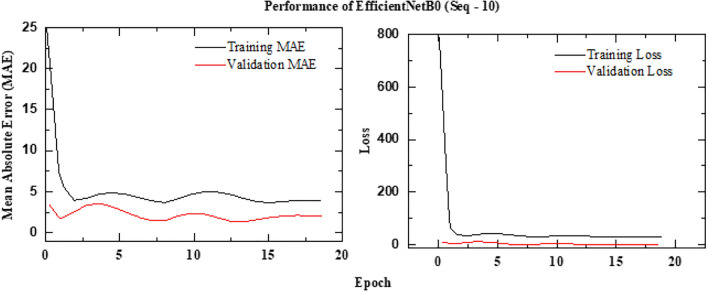
MAE Performance of EfficientNet Seq10 framework.

The schematic architecture layout of EfficientNet-B0 framework is shown in Figure 6 and its implementation is shown with the help of Algorithm 1. The predictive performance of the EfficientNet Seq5 (using the sequence of five continuous images) and Seq10 (using the sequence of ten continuous images) frameworks is presented in Figure 7 and Figure 8, respectively. The observed leakage prediction for the EfficientNet Seq5 framework is 19.30%, compared to the actual leakage of 17.55%, while for the EfficientNet Seq10 framework, the observed prediction leakage is 34.03% against an actual leakage of 29.88%. The corresponding Mean Absolute Error (MAE) performance is illustrated in Figure 9 and Figure 10 for the EfficientNet Seq5 and Seq10 frameworks, respectively.

**Algorithm 1:**
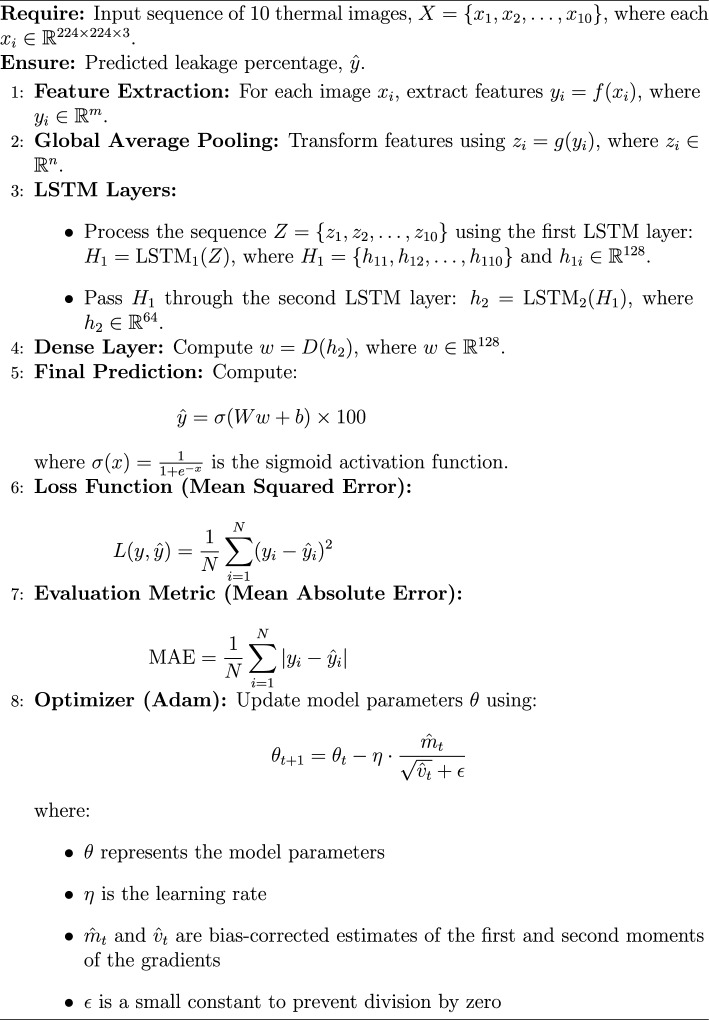
EfficientNetB0 Architecture for Leakage Detection

### Performance of CNN-LSTM framework

**Fig. 11:**
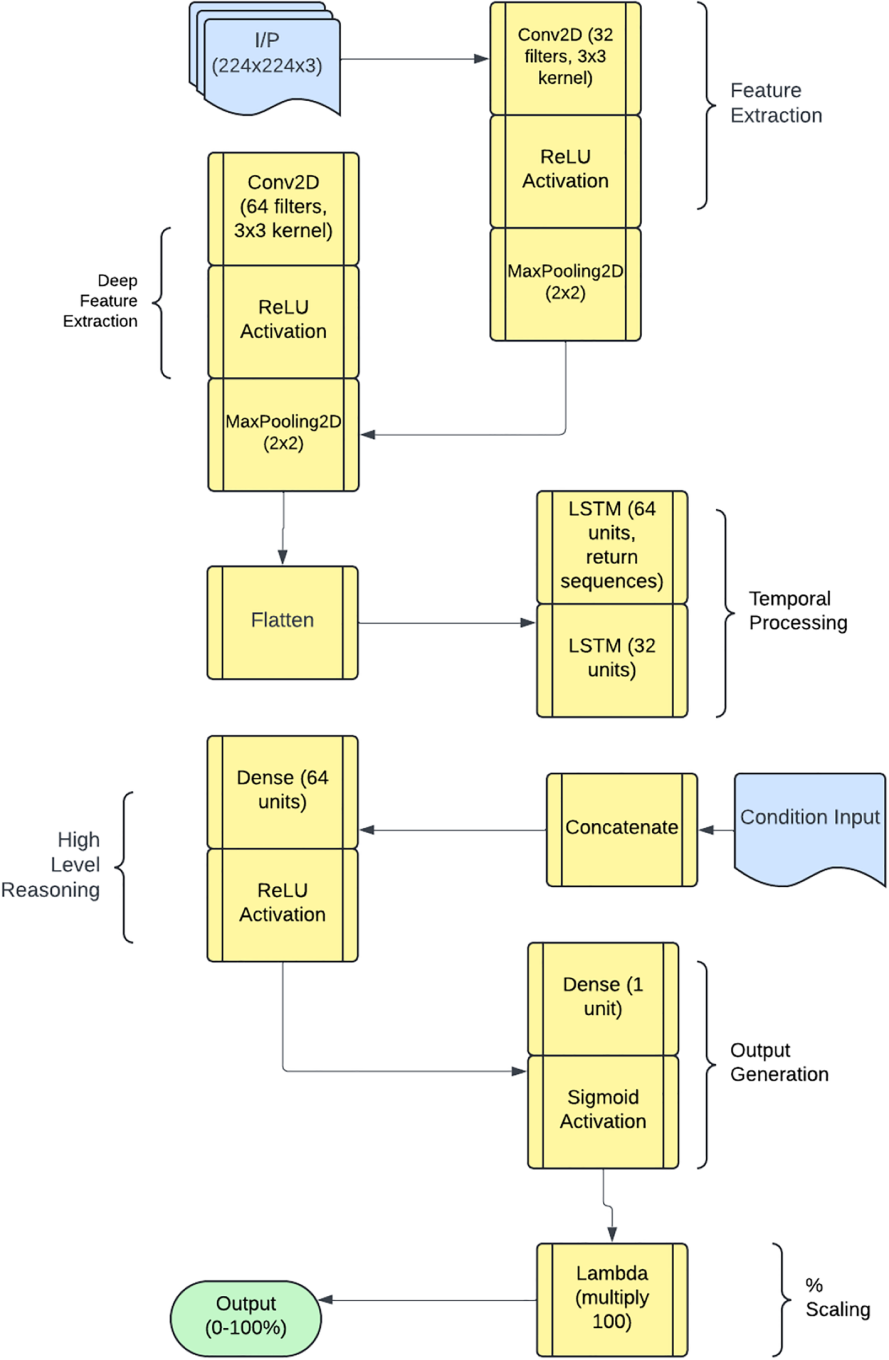
Architecture of the CNN-LSTM framework.

**Fig. 12:**
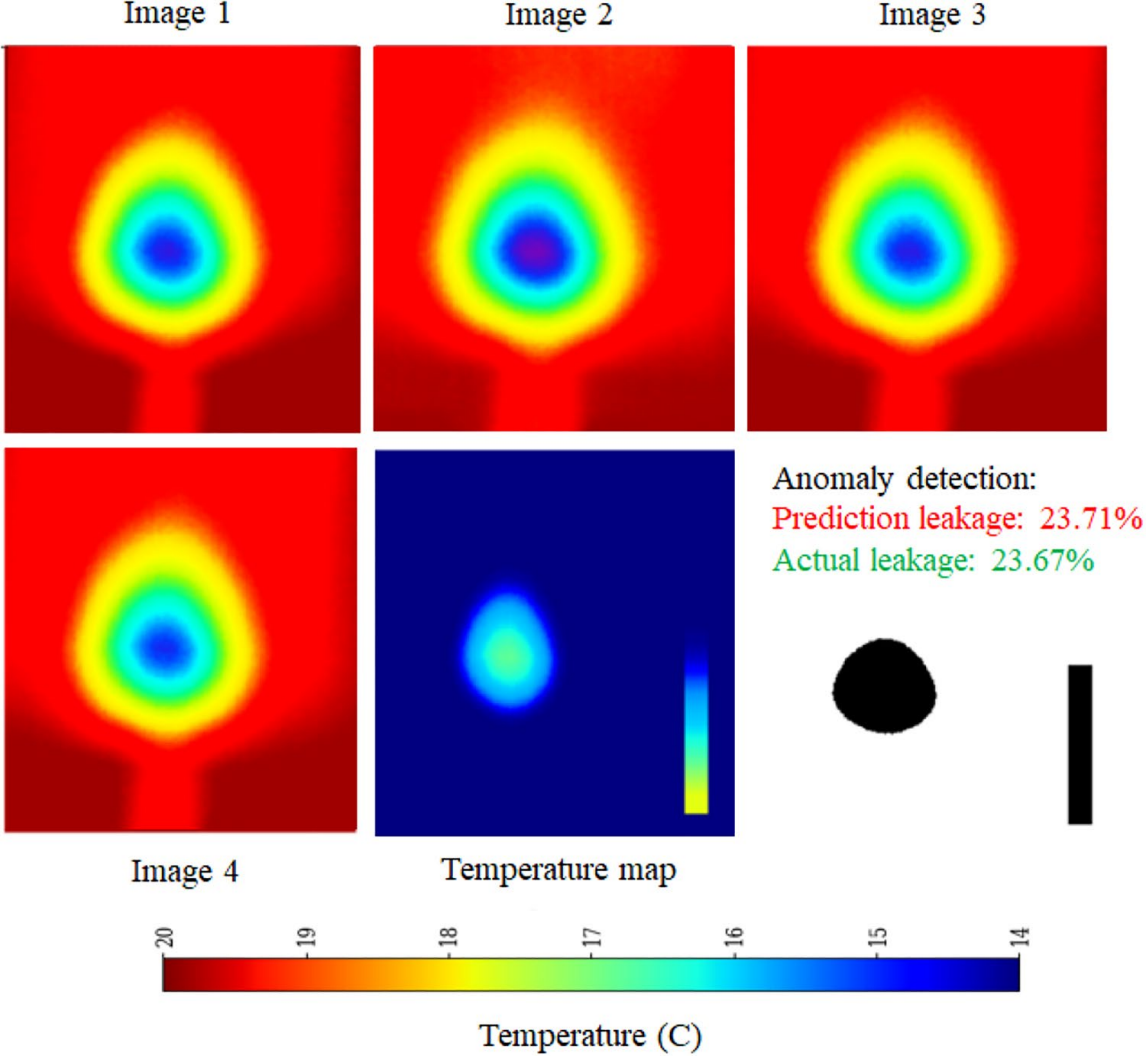
Performance of CNN-LSTM framework.

**Fig. 13:**
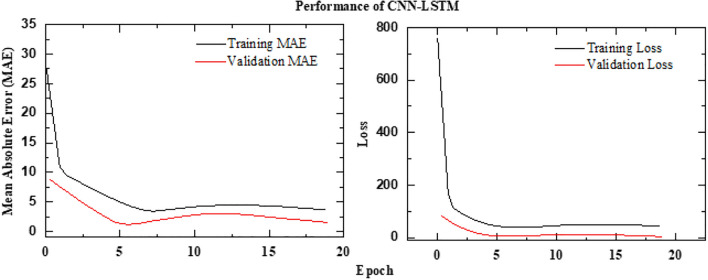
MAE Performance of CNN-LSTM framework.

The implementation of the proposed framework follows a structured deep-learning approach. It begins with a simple Convolutional Neural Network (CNN) as the base feature extractor, consisting of two Conv2D layers with MaxPooling for spatial feature extraction. To efficiently process sequential image data, a time-distributed CNN is employed, ensuring consistent feature extraction across frames. The extracted features are then passed through two Long Short-Term Memory (LSTM) layers with 64 and 32 units, respectively, enabling the model to capture temporal dependencies. A fully connected dense layer with 64 units and ReLU activation further refine the learned representations. Finally, the model outputs a single-unit dense layer with a sigmoid activation function, scaling the predictions within the range of 0 to 100%. This architecture effectively combines spatial and temporal feature learning, making it well-suited for image sequence analysis. The schematic architecture layout of CNN-LSTM framework is shown in Figure 11 and its implementation is shown with the help of Algorithm 2. The predictive performance of the CNN-LSTM frameworks is presented in Figure 12. The observed leakage prediction for the CNN-LSTM framework is 23.71%, compared to the actual leakage of 23.67%. The corresponding Mean Absolute Error (MAE) performance is illustrated in Figure 13.

**Algorithm 2:**
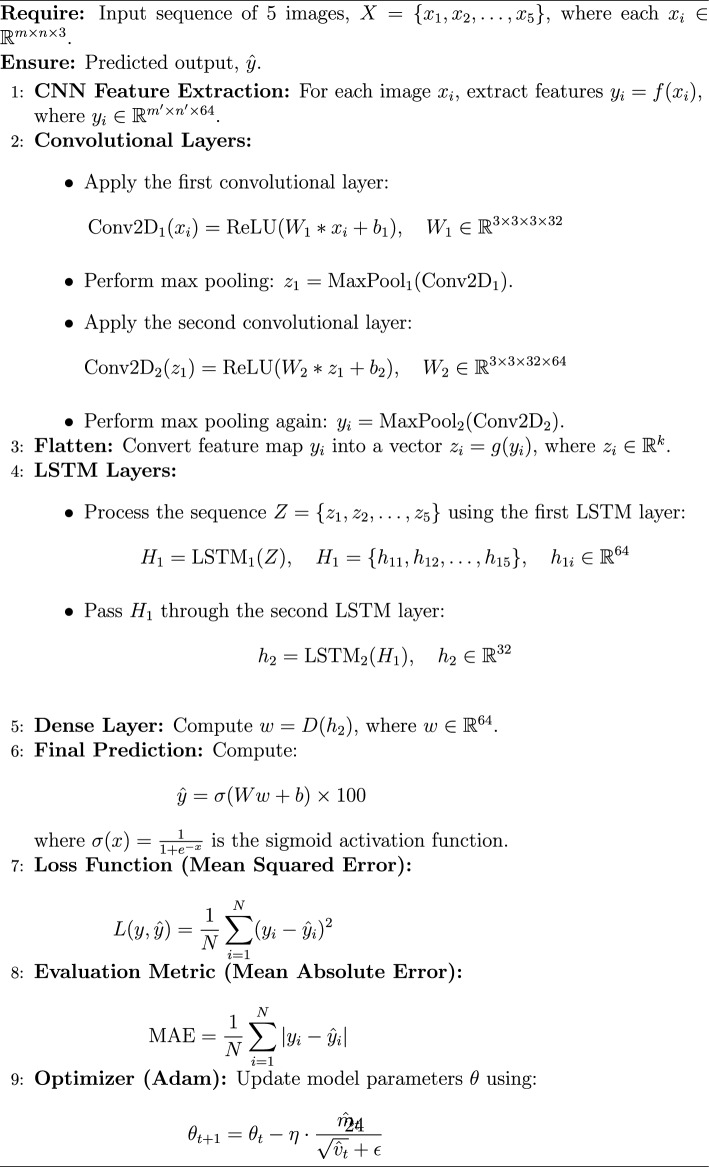
CNN-LSTM Model for Thermal Leakage Detection

### Performance of ResNet50 framework

**Fig. 14:**
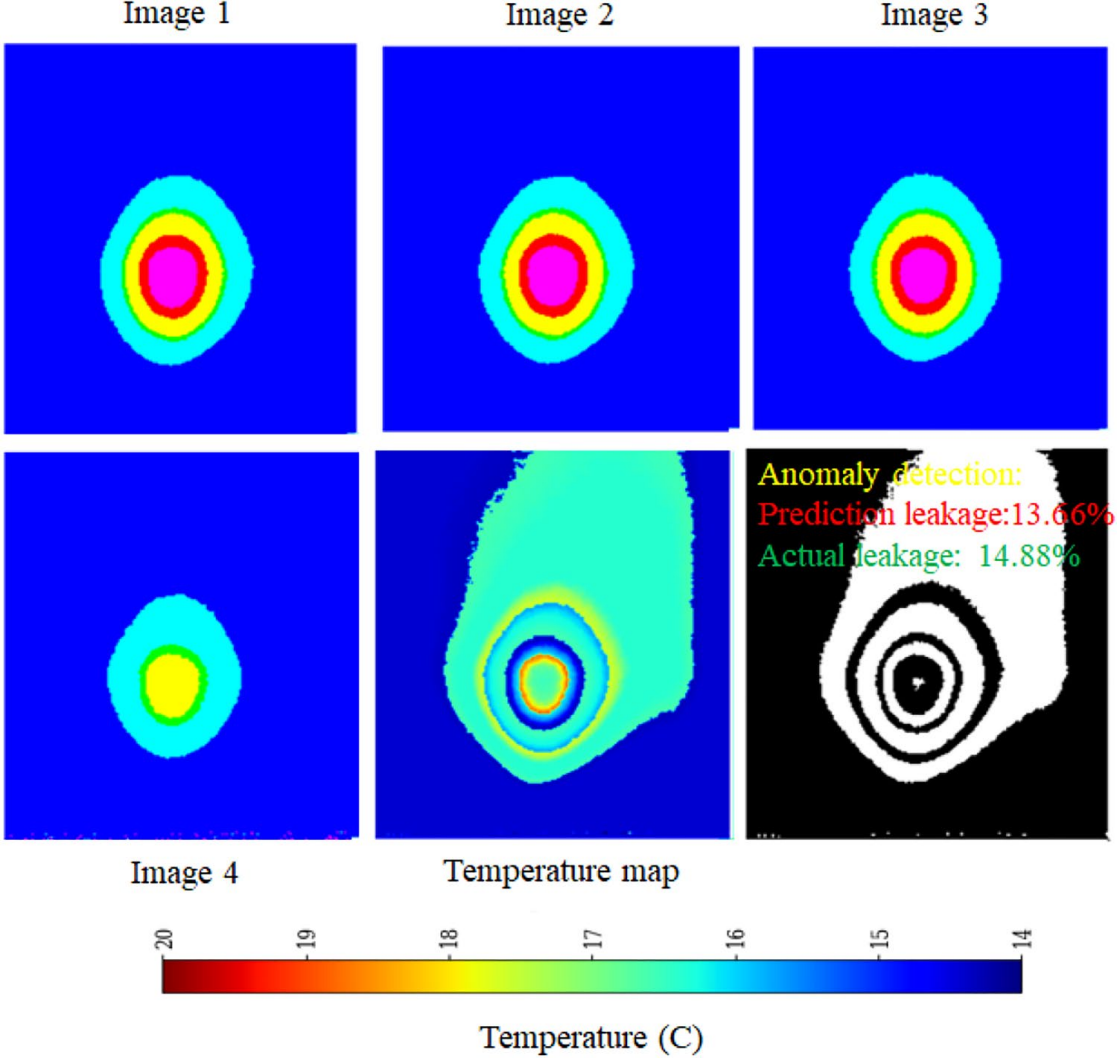
Performance of ResNet framework.

**Fig. 15:**
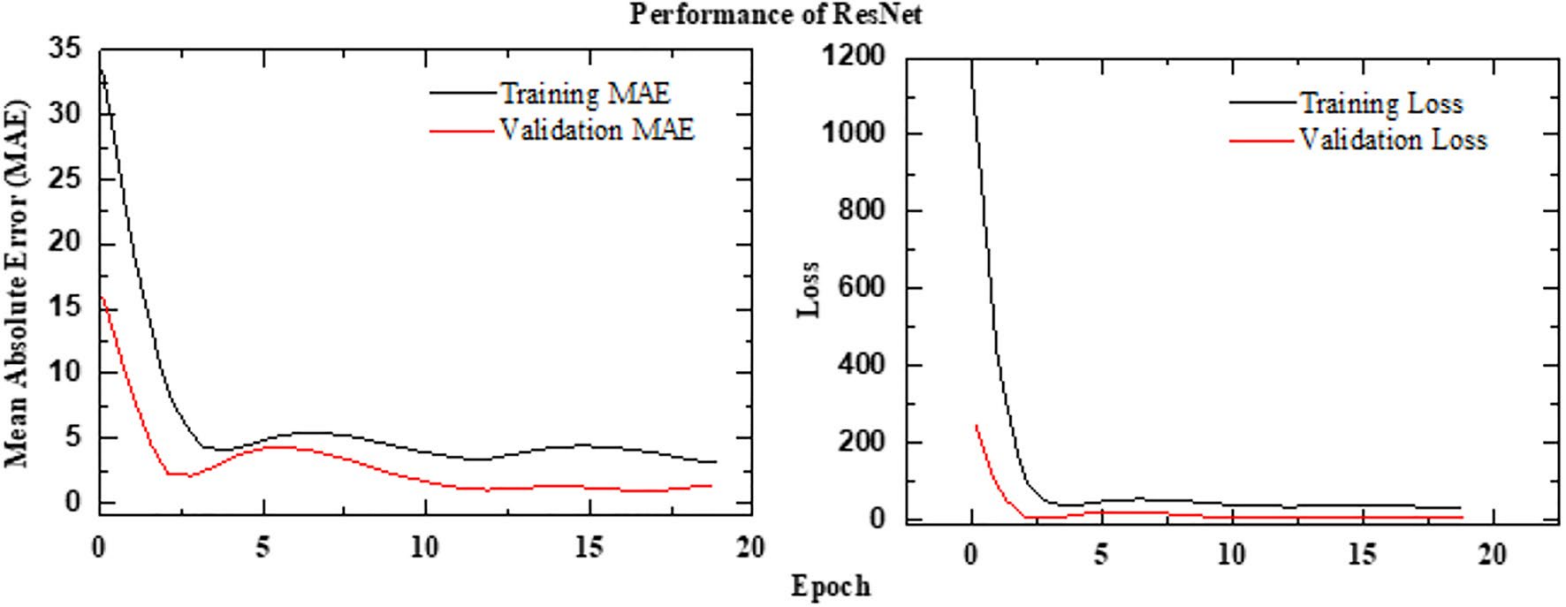
MAEPerformance of ResNet framework.

Residual Networks, introduced by He et al.^34^, address the problem of training very deep neural networks by introducing skip connections. This innovation allows for the creation of much deeper and more powerful models for image recognition and has become a fundamental building block in many modern architectures^34^. The proposed framework is implemented using a deep learning architecture that integrates both spatial and temporal feature extraction. At its core, ResNet50 serves as the base feature extractor, utilizing pretrained ImageNet weights while excluding the top layers. A Global Average Pooling 2D (GAP) layer follows to reduce spatial dimensions while preserving essential features. To efficiently process sequential image data, a time-distributed ResNet50 is employed, ensuring consistent feature extraction across frames. The extracted features are then passed through two Long Short-Term Memory (LSTM) layers with 128 and 64 units, respectively, to capture temporal dependencies. A fully connected dense layer with 128 units and ReLU activation further refines the learned representations. Finally, the model produces a single-unit dense layer with a sigmoid activation function, scaling the predictions within the range of 0 to 100%. This architecture effectively combines the powerful feature extraction capabilities of ResNet50 with LSTM-based temporal modeling, making it well-suited for sequential image analysis. The schematic architecture implementation is shown with the help of Algorithm 3. The predictive performance of the ResNet frameworks is presented in Figure 14. The observed leakage prediction for the CNN-LSTM framework is 13.06%, compared to the actual leakage of 14.88%. The corresponding Mean Absolute Error (MAE) performance is illustrated in Figure 15.

**Algorithm 3:**
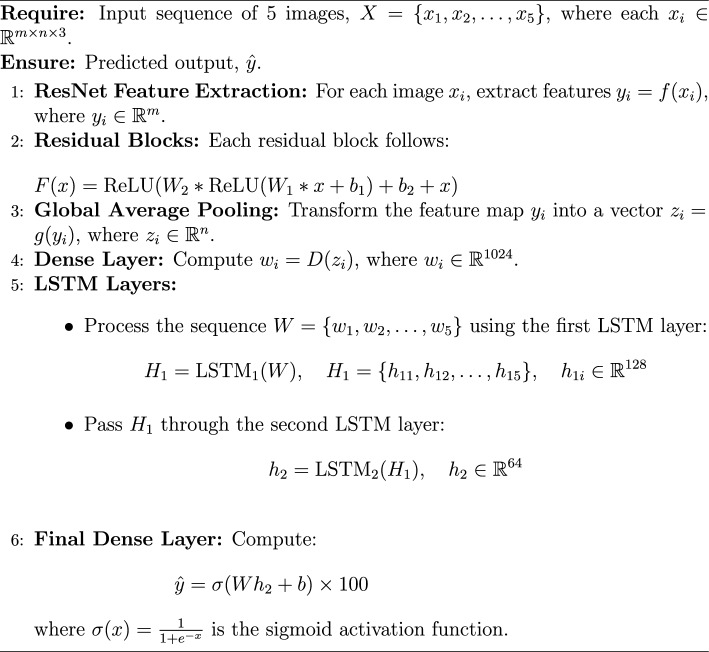
ResNet Model for Thermal Leakage Detection

### Performance of AlexNet framework

AlexNet is a pioneering deep convolutional neural network (CNN) architecture developed by Krizhevsky et al.^35^. It significantly outperformed previous methods in the ImageNet Large Scale Visual Recognition Challenge, marking a turning point in the adoption of deep learning for computer vision tasks^35^

**Fig. 16:**
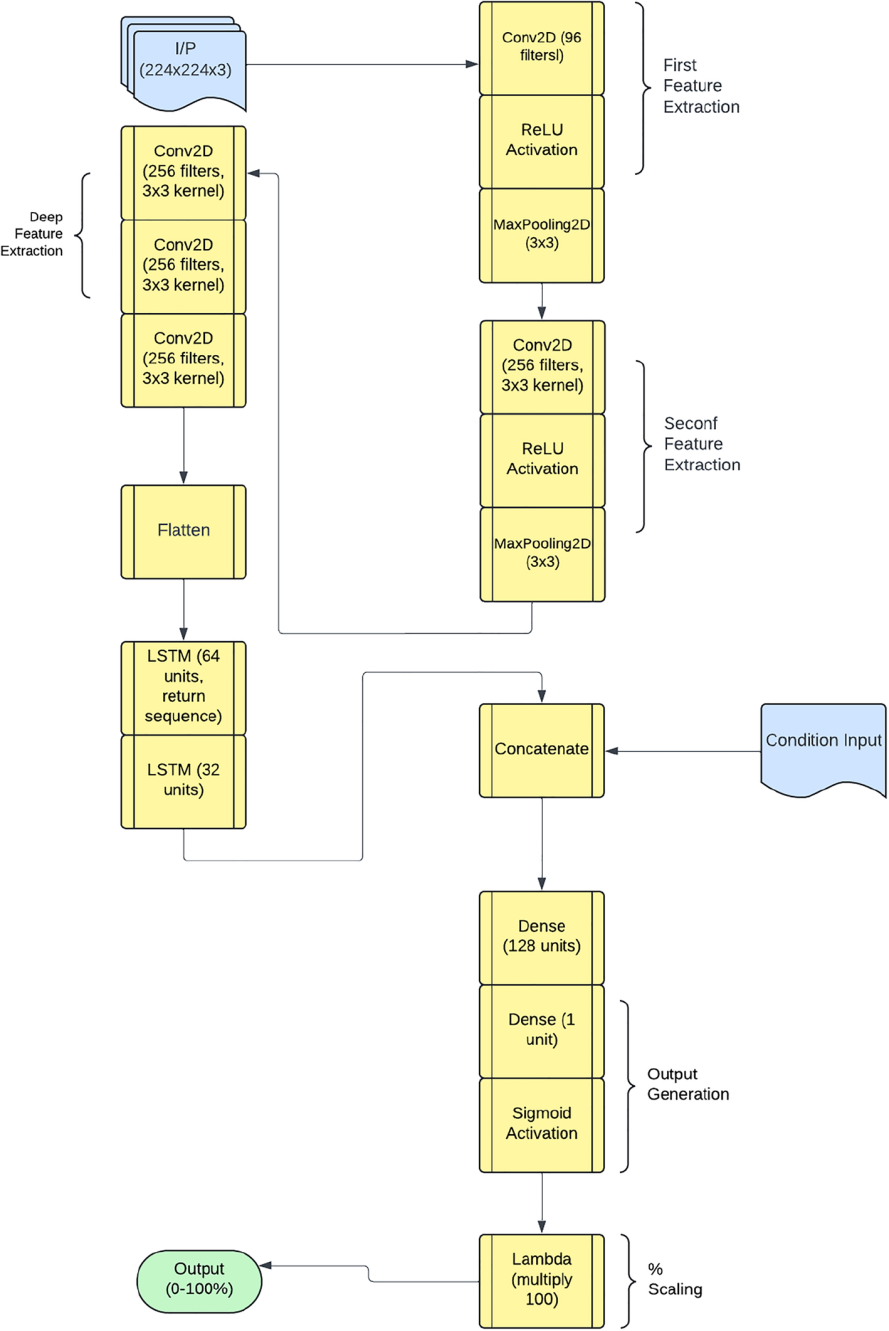
Architecture of the AlexNet framework.

**Fig. 17:**
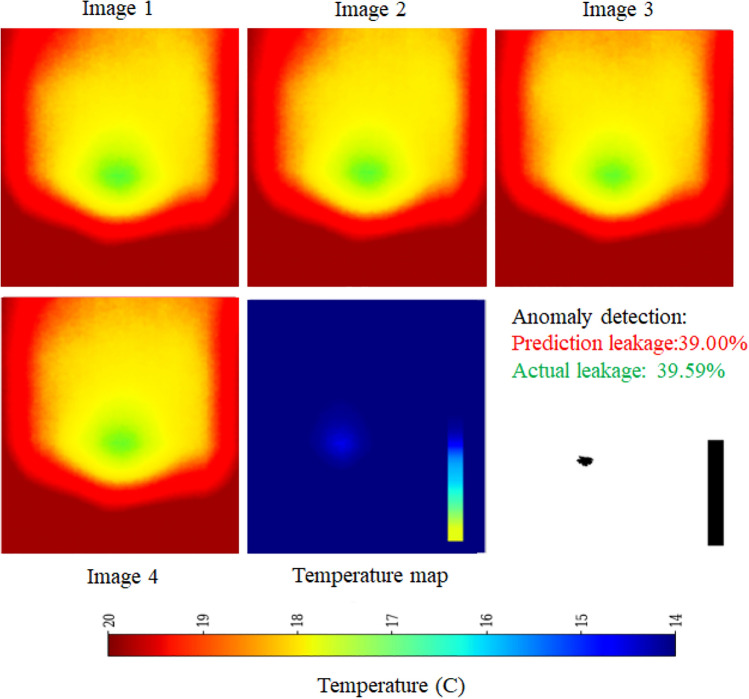
Performance of AlexNet framework.

**Fig. 18:**
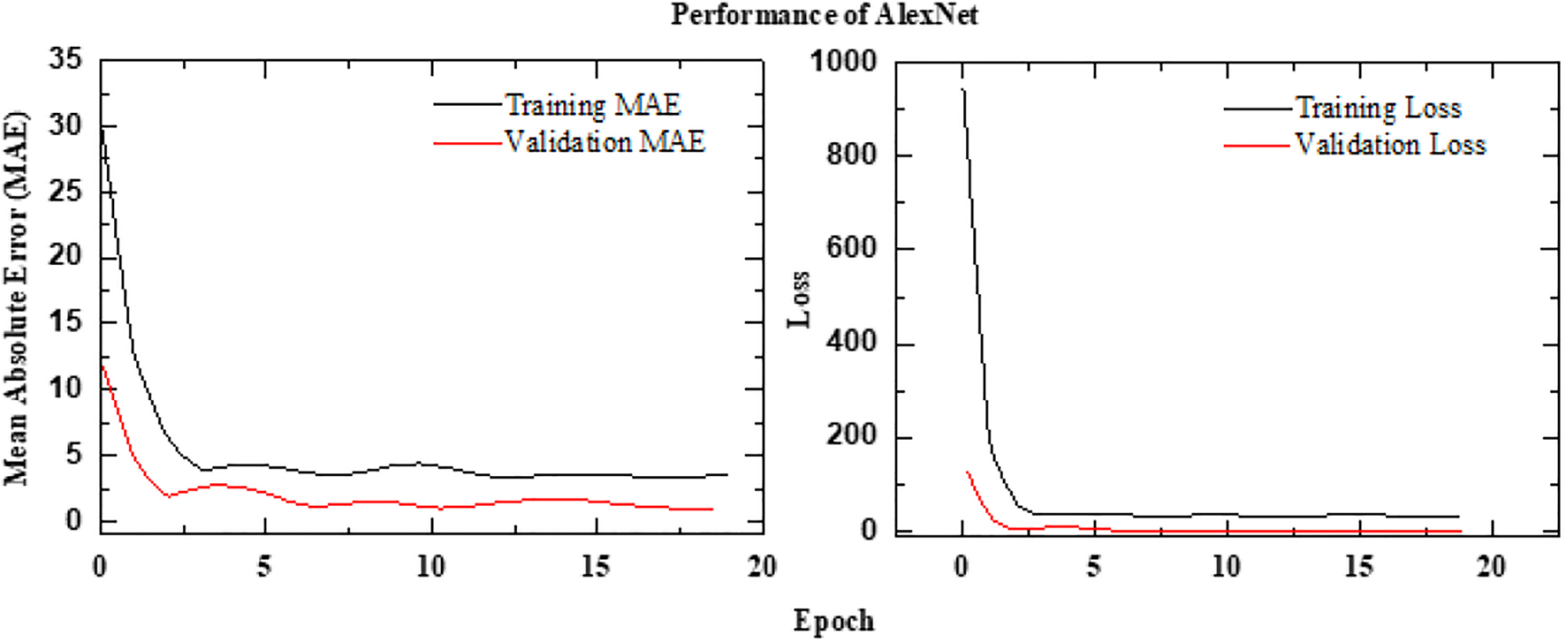
MAE Performance of AlexNet framework.

The proposed framework is implemented using a deep learning architecture that integrates spatial and temporal feature extraction. It utilizes an AlexNet-inspired CNN as the base feature extractor, trained with custom weights to optimize performance for the given task. To handle sequential image data efficiently, a time-distributed AlexNet-based CNN is employed, ensuring consistent spatial feature extraction across frames. The extracted features are then processed through two Long Short-Term Memory (LSTM) layers with 128 and 64 units, respectively, enabling the model to capture temporal dependencies effectively. A fully connected dense layer with 128 units and ReLU activation further refines the learned representations. Finally, the model produces a single-unit dense layer with a sigmoid activation function, scaling the predictions within the range of 0 to 100%. This architecture leverages the feature extraction power of AlexNet while integrating LSTM-based temporal modeling, making it well-suited for sequential image analysis. The schematic architecture is shown in Figure 16 and its implementation is shown with the help of Algorithm 4. The results are shown in Figure 17. The observed leakage prediction for the AlexNet framework is 39.00%, compared to the actual leakage of 39.59%. The corresponding Mean Absolute Error (MAE) performance is illustrated in Figure 18.

**Algorithm 4:**
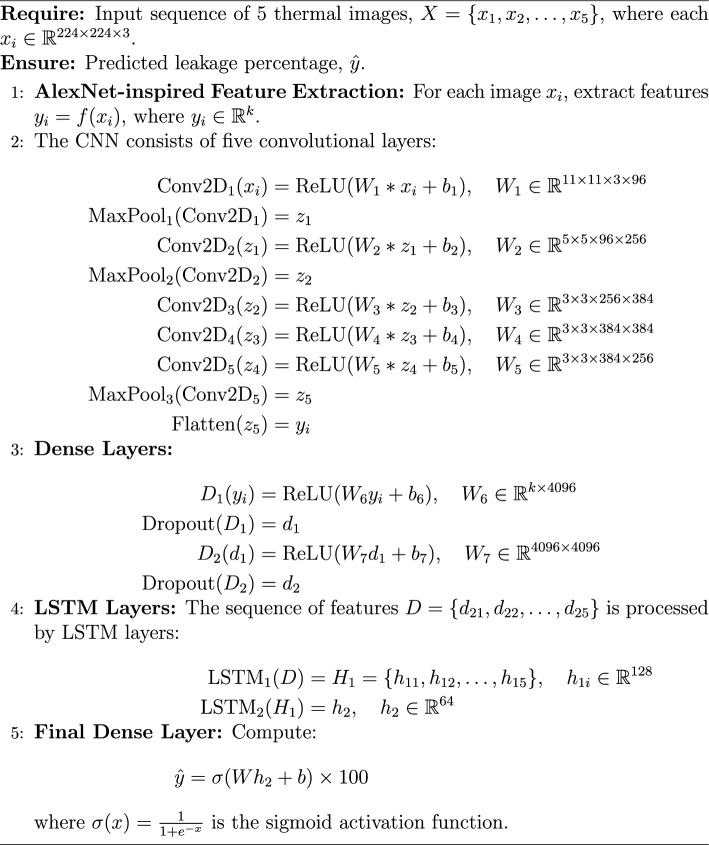
AlexNet-inspired Architecture for Thermal Image Leakage Detection

### Comparative performance of all frameworks under different testing conditions

The comparative analysis of deep learning frameworks for seepage and ponding prediction under various environmental conditions, as presented in Table 4, evaluates the alignment between predicted and actual values, where lower deviations signify superior predictive accuracy. Among the examined DL frameworks, ResNet demonstrates the highest reliability, particularly excelling in scenarios involving clear ponding and ponding under rainfall, with predicted values (21.13% and 22.65%) closely matching actual measurements (19.6% and 21.97%). Whereas, EfficientNet Seq5 shows strong predictive performance in most conditions, though it slightly underestimates clear ponding (actual: 19.6%, predicted: 18.35%) and ponding under rainfall (actual: 21.97%, predicted: 20.15%). EfficientNet Seq10, while generally accurate, also underestimates dry and clean surface conditions (actual: 20.48%, predicted: 18.91%) and clear ponding (actual: 19.6%, predicted: 18.01%). CNN-LSTM performs consistently well across conditions, particularly in dry and clean surface scenarios, though it underestimates ponding under rainfall (actual: 21.97%, predicted: 20.38%). AlexNet maintains competitive accuracy, with minor overestimations in clear ponding (actual: 19.6%, predicted: 20.29%) and moderate variations in ponding under rainfall (actual: 21.97%, predicted: 20.82%). In summary, these results highlight the importance of selecting a DL framework that effectively captures the underlying seepage and ponding extent while minimizing predictive errors. ResNet emerges as the most reliable choice for complex seepage and ponding conditions, followed by CNN-LSTM for its stable performance across various conditions. EfficientNet models, while strong in predictive capability, exhibit a tendency to underestimate key environmental factors, suggesting potential room for optimization. AlexNet, though competitive, shows slightly higher variations that may impact its robustness in real-world applications. The findings of this study reinforce the necessity of deploying robust deep learning architectures to enhance seepage and ponding prediction accuracy, which is crucial for early detection and mitigation strategies in earthen embankments.

**Table 4:** Comparative performance of all DL frameworks under different environmental conditions.

Deep Learning (DL)		Performance under different conditions on D/S					
Framework		Rainfall over	Dry	Dry and clean	Clean surface	Clear	Ponding under
		vegetation (%)	vegetation (%)	surface (%)	under rainfall (%)	ponding (%)	rainfall (%)
EfficicentNet Seq5	Actual	34.88	8.96	21.27	10.62	19.6	21.97
	Prediction	34.17	8.67	18.21	11.28	18.35	20.15
EfficicentNet Seq10	Actual	33.93	9.28	20.48	10.61	19.6	21.97
	Prediction	34.03	9.1	18.91	11.94	18.01	20.95
CNN-LSTM	Actual	34.88	8.96	21.27	10.62	19.6	21.97
	Prediction	35.76	8.59	21.0	10.31	17.95	20.38
ResNet	Actual	34.88	8.96	21.27	10.62	19.6	21.97
	Prediction	34.65	9.25	18.79	11.42	21.13	22.65
AlexNet	Actual	34.88	8.96	21.27	10.62	19.6	21.97
	Prediction	34.85	9.35	18.82	10.47	20.29	20.82

## Conclusion

This study systematically evaluated the performance of various deep learning architectures for leakage detection using thermal imagery sequences, with model accuracy assessed via the Mean Absolute Error (MAE) metric. The Modified AlexNet with a sequence length of 5 demonstrated great predictive capability, achieving the lowest MAE of 1.02%, closely followed by EfficientNet (1.16%) and ResNet (1.29%). The CNN model, though simpler, attained a relatively higher MAE of 2.06%, reaffirming the advantage of deeper and more complex architectures. Notably, increasing the sequence length to 10 in EfficientNet resulted in a significant drop in performance, with the MAE rising to 3.20%. This suggests that longer temporal sequences may introduce excessive noise or redundant information, potentially leading to overfitting or reduced generalization. The consistently strong performance of models with a sequence length of 5 highlights its effectiveness in capturing essential temporal leakage patterns while avoiding unnecessary complexity. Despite its superior accuracy, the steeper gradient of the validation MAE curve in Modified AlexNet suggests potential limitations in scalability. EfficientNet and ResNet, due to their balanced accuracy and stability, appear to be more suitable for larger and more diverse datasets. These findings underscore the importance of selecting the appropriate combination of model architecture and sequence length to optimize performance. Additionally, the study demonstrates the readiness of these models for real-world applications, such as industrial leak detection, pipeline monitoring, and infrastructure maintenance, where early and accurate leakage identification is crucial. Future work should explore attention mechanisms or transformer-based models to enhance temporal feature extraction. Moreover, integrating multi-modal sensor data, including acoustic and pressure sensors, could further improve detection reliability. Interpretability techniques should also be developed to gain deeper insights into model predictions, ultimately leading to more transparent and effective leakage detection frameworks. The major takeaway from the presented studies are as follows:

- The modified AlexNet (Seq5) recorded the lowest MAE of 1.02%, making it the most precise model. However, ResNet demonstrated the highest stability and consistency across various testing conditions.
- Both EfficientNet (Seq5) and ResNet exhibited competitive performance, with strong generalization capabilities reflected in MAE values of 1.16% and 1.29%, respectively. Notably, ResNet outperformed other models in terms of scalability.
- Extending the sequence length to 10 in EfficientNet resulted in a substantial performance decline (MAE = 3.20%), highlighting the adverse effects of excessive temporal information.
- A sequence length of 5 proved to be optimal, effectively balancing temporal feature extraction with model stability.
- Future improvements may involve integrating transformer-based models or hybrid deep learning frameworks to enhance temporal and spatial feature extraction. Additionally, incorporating multi-modal sensor data, such as pressure and acoustic sensors, could further improve prediction reliability. Interpretability techniques, such as explainable AI methods, should be explored to gain deeper insights into the decision-making process of these models, ultimately leading to more effective predictive frameworks for seepage and ponding detection.

## Data Availability

The datasets used and/or analysed during the current study are available from the corresponding author on reasonable request.
